# Quadruple Target Evaluation of Diversity-Optimized Halogen-Enriched Fragments (HEFLibs) Reveals Substantial Ligand Efficiency for AP2-Associated Protein Kinase 1 (AAK1)

**DOI:** 10.3389/fchem.2021.815567

**Published:** 2022-02-02

**Authors:** Marcel Dammann, Markus Kramer, Markus O. Zimmermann, Frank M. Boeckler

**Affiliations:** ^1^ Lab for Molecular Design and Pharmaceutical Biophysics, Department of Pharmacy and Biochemistry, Institute of Pharmaceutical Sciences, Eberhard Karls Universität Tübingen, Tübingen, Germany; ^2^ Institute of Organic Chemistry, Eberhard Karls Universität Tübingen, Tübingen, Germany; ^3^ Interfaculty Institute for Biomedical Informatics (IBMI), Eberhard Karls Universität Tübingen, Tübingen, Germany

**Keywords:** halogen bonding, fragment-based drug discovery, saturation transfer difference, isothermal titration calorimetry, DOT1L, CAMK1G, AAK1, IDO1

## Abstract

Fragment-based drug discovery is one of the most utilized approaches for the identification of novel weakly binding ligands, by efficiently covering a wide chemical space with rather few compounds and by allowing more diverse binding modes to be found. This approach has led to various clinical candidates and approved drugs. Halogen bonding, on the other hand, has gained traction in molecular design and lead optimization, but could offer additional benefits in early drug discovery. Screening halogen-enriched fragments (HEFLibs) could alleviate problems associated with the late introduction of such a highly geometry dependent interaction. Usually, the binding mode is then already dominated by other strong interactions. Due to the fewer competing interactions in fragments, the halogen bond should more often act as an anchor point for the binding mode. Previously, we proposed a fragment library with a focus on diverse binding modes that involve halogens for gaining initial affinity and selectivity. Herein, we demonstrate the applicability of these HEFLibs with a small set of diverse enzymes: the histone-lysine N-methyltransferase DOT1L, the indoleamine 2,3-dioxygenase 1 (IDO1), the AP2-associated protein kinase 1 (AAK1), and the calcium/calmodulin-dependent protein kinase type 1G (CAMK1G). We were able to identify various binding fragments *via* STD-NMR. Using ITC to verify these initial hits, we determined affinities for many of these fragments. The best binding fragments exhibit affinities in the one-digit micromolar range and ligand efficiencies up to 0.83 for AAK1. A small set of analogs was used to study structure-affinity relationships and hereby analyze the specific importance of each polar interaction. This data clearly suggests that the halogen bond is the most important interaction of fragment 9595 with AAK1.

## Introduction

Throughout the past decade, fragment-based drug discovery (FBDD) rose to one of the standard approaches of early drug discovery (Jacquemard and Kellenberger, 2019). This is illustrated by the number of clinical candidates derived from fragment screening in the pharmaceutical industry (Jahnke et al., 2020). Based on the efficient representation of chemical space by fragment libraries, the size of a typical fragment library can be significantly more restricted than those libraries used for high throughput-screening (HTS) (Murray and Rees, 2009). In addition, academic groups have been grown fond of the FBDD as well, mainly because of the relatively low threshold in creating, procuring, and maintaining such a library, in comparison to the enormous resources necessary for a typical HTS library formed by drug-like compounds (Konteatis, 2021). In a previous publication, we designed and established a specialized fragment library, followed by an experimental characterization of its fragment solubility (Heidrich et al., 2019). This library is distinguishable from others by the extensive enrichment of halogen atoms and their integration in a broad diversity of binding motifs. We set out to improve and expand the use of halogen atoms in drug discovery, to explore and utilize the unconventional binding modes of halogens in comparison with the classical interactions exhibited by the more frequently used atoms in drug-like molecules (i.e., C, O, N, S). Due to their increasing anisotropic electron distribution, chlorine, bromine, and iodine atoms can engage in halogen bonding (XB), which is highly directional and shows strict geometrical dependence of attractiveness (Wilcken et al., 2012a). In drug discovery, a halogen bond is usually rationalized by the σ-hole concept (Clark et al., 2007). This term is coined based on the electron deficient region in the elongation of the carbon-halogen bond (Politzer et al., 2013; Cavallo et al., 2016). The σ-hole describes the potential to form attractive interactions with electron rich regions of various substructures in the binding site, the most frequent being the backbone carbonyl of each amino acid (Wilcken et al., 2012b; Zimmermann et al., 2015; Zimmermann and Boeckler, 2016; Lange et al., 2019). Electron rich sidechains (e.g., aspartate, glutamate) (Zimmermann et al., 2016), serine, histidine (Lange et al., 2013), or methionine (Wilcken et al., 2011)) are suitable halogen bond donors in proteins and can be expanded to nucleotides (Ho, 2017). The best interaction energy is typically seen at the C-X-O angle of 180° ± 30° and a distance between halogen and electron donor of 3–4 Å (Wilcken et al., 2012b). These conditions enable a better selectivity profile for halogen bearing compounds (Wilcken et al., 2013). Typically, halogens are incorporated during the hit-to-lead or lead optimization stage of drug discovery. Still, because of their high directionality, molecular design of halogen bonds is not trivial. While expecting that the affinities improve from chlorine to bromine and from bromine to iodine, halogen exchange can lead to surprising plateaus of affinity (Lange et al., 2015; Heroven et al., 2018). In addition, it has been shown that protein stability and enzyme activity can be modulated or improved by protein engineering of halogen bonds (Carlsson et al., 2018).

A promising fragment typically shows at maximum three possible hydrogen bond donors or acceptors (Giordanetto et al., 2019; Konteatis, 2021). Thus, statistically, a halogen bond formed by a fragment competes with fewer polar interactions. Starting with halogen enriched fragments (Wilcken et al., 2012a; Heidrich et al., 2019) increases the chance of generating unusual binding poses, where a halogen bond is essential for the binding mode of the initial hit. In addition, we propose to improve our understanding of strong halogen bonding motifs through this strategy and learn more about privileged features for accepting halogen bonds in the protein environment.

Herein, we show the first characterization of the diversity-optimized library of HEFLibs (Heidrich et al., 2019) with a stability assessment of potentially reactive fragments followed by experimental screening of a small subset of diverse targets (namely, DOT1L, IDO1, AAK1, and CAMK1G). Eventually, we evaluate the hit fragments by direct ITC as a validation step. We were able to show comparable hit-rates of our library with other libraries (Keseru et al., 2016) and were able to find binding fragments with exceptional ligand efficiency. A small set of analogs was tested to elucidate the importance of the distinctive features of the best hit.

**FIGURE 1:**
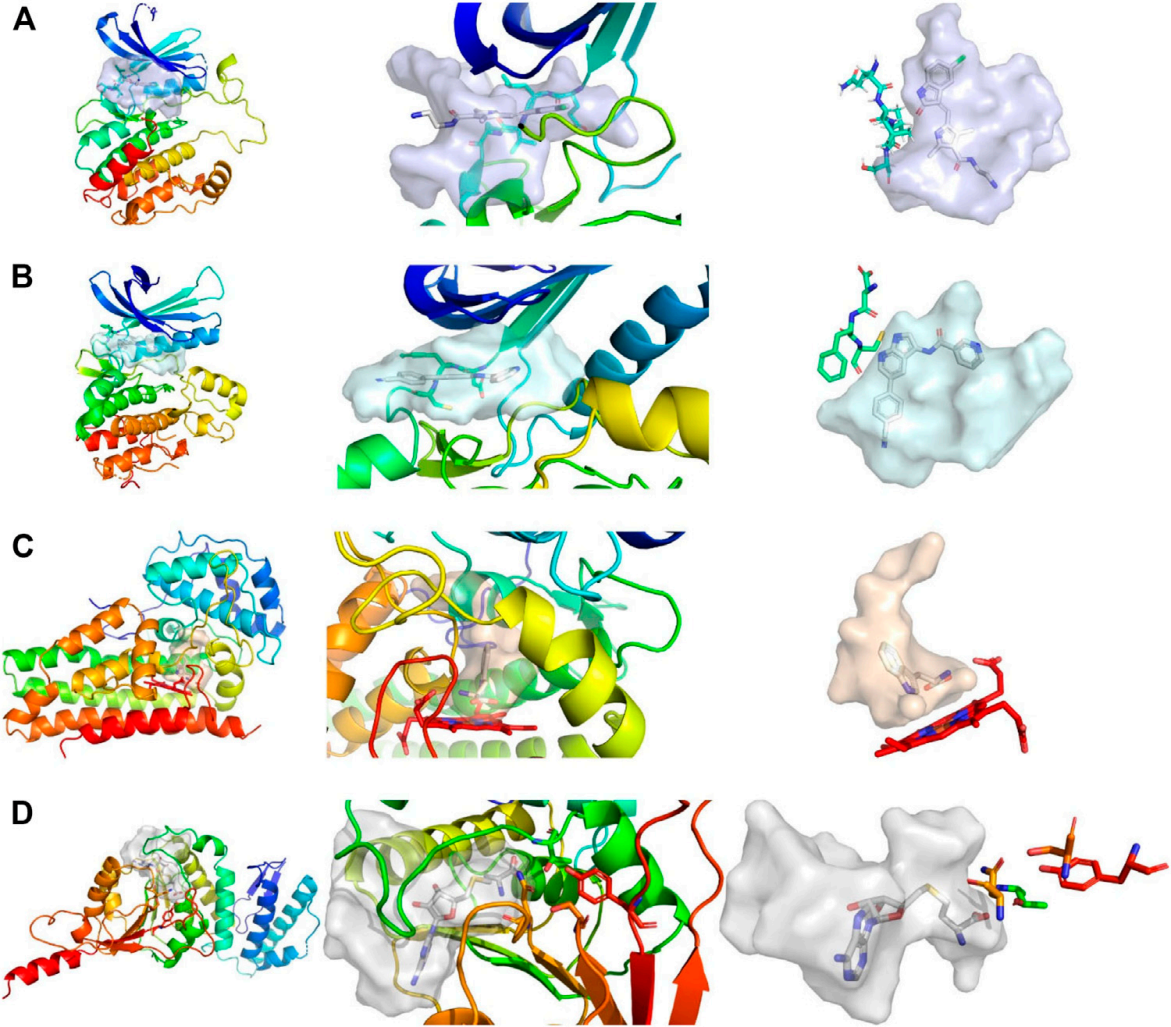
Comparison of active sites, from left to right, shows the complete protein, a close-up view of the pocket, and the shape of the pocket with characteristic residues explicitly depicted. **(A)** Structure of CAMK1G (PDB entry 2JAM), where residues of the hinge region are shown. **(B)** Structure of AAK1 (PDB entry 5L4Q) with residues shown that are part of the hinge region. **(C)** Structure of IDO1 (PDB entry 6E46) shown with the JK-(gate)-loop colored in red in the middle. The heme cofactor and tryptophan are depicted as a reference. **(D)** Structure of DOT1L (PDB entry 3QOX), where the residues depicted are vital for methylation of the lysine. S-Adenosyl homocysteine is depicted in the pocket. The kinase pockets are the most voluminous, followed by DOT1L and IDO1. The IDO1 pocket is the most enclosed pocket, which changes depending on the heme binding state from the JK-loop. Surfaces were created by filling the binding site with a 1 Å spaced grid and by deleting every grid point within 2.5 Å of any protein atom. The figure was prepared with PyMOL 2.3.3.

While the library was designed to feature diverse halogen bonding interfaces, here, we want to focus on the diversity of active sites on the target proteins. We chose these proteins based on their relevance in cancer pathogenesis, the level of experimental knowledge about the protein target, and the heterogenicity of the structural features and interaction potential of the binding pocket (see Figure 1). DOT1L was already an established fragment-based drug discovery target, enabling a direct comparison with published data (Chen et al., 2016; Mobitz et al., 2017). IDO1 was chosen for its cofactor-featuring binding pocket, which binds tryptophan as a substrate (Coletti et al., 2017; Nelp et al., 2018). Based on established series of IDO modulators, we expect that the cofactor heme is a potential interaction hotspot. In addition, the binding pocket is prone to recognize (hetero)aromatic ring systems, which present the main scaffold for all of our fragments. AAK1 and CAMK1G are both kinases. While even less is known about CAMK1G compared to AAK1, both kinases belong to the lesser studied part of the kinome. This becomes more obvious by the scarcity of structures (Hu et al., 2015). Therefore, a possible tool compound, generated from an initial hit, could be beneficial for the understanding of the involved kinase pathways. Some descriptors related to structure and interactions, characterizing the active site of the four proteins, are compared in Table 1: volume [Å^3^], surface area [Å^2^], polar surface area (PSA) [Å^2^], percentage of PSA, and the number of negative and positive amino acids. Based on this comparison, the active site of CAMK1G contains the highest number of charged amino acids and is significantly larger than the others. AAK1, on the other hand, has a clearly lower volume while maintaining a similar surface, with a high PSA percentage. The IDO1 has by far the smallest active site (with the heme factor present) and the least %PSA and only two charged amino acids. The active site of DOT1L is comparable in size with the AAK1, but has substantially smaller surface area and a higher net negative charge. These metrics portray the diversity in the target selection, but do not account for possible allosteric pockets or alternative conformations, which have already been described for all four proteins (Scheufler et al., 2016; Nelp et al., 2018; Yueh et al., 2019).

**TABLE 1:** Comparison of binding site metrics: Volume was measured as defined above. The van der Waals radii were used for surface calculations in PyMOL 2.3.3 Polar surface area (PSA) and %PSA were calculated as defined in PyMOL 2.3.3 Positively and negatively charged amino acids (AA) are counted based on standard assumptions of the protonation state at physiological pH. Thus, histidine is not considered as positively charged by default.

	CAMK1G	AAK1	IDO1	DOT1L
Volume [Å^3^]	622	334	169	316
Surface area [Å^2^]	1,050.3	967	325.1	766.6
PSA [Å^2^]	477.9	479.7	99.2	342.6
% PSA	45.5	49.6	30.5	44.7
Neg. charged AA	6	3	1	4
Pos. charged AA	3	2	1	1

## Methods

### GSH Stability Assessment

The performed GSH-stability assay for the fragments was based on an established protocol developed for heterocyclic electrophilic fragments (Keeley et al., 2019). A 500 µM fragment solution in PBS buffer pH 7.4, 10% acetonitrile and 200 µM indoprofen as an internal standard, was added to 10 mM GSH solution in a 1:2 ratio. The reaction temperature was 40°C. The mixture was analyzed by HPLC (Column: Phenomenex Kinetex 2.6 µm C8 100 Å 150 × 4.6 mm) with an injection volume of 5 μl and a flow rate of 0.5 ml/min at 23°C after a reaction duration of 0, 1, 2, 4, 6, and 24 h. The reaction of fragments with GSH was determined by measuring the decreasing area under the curve (AUC) of the compound relative to the internal standard indoprofen. The gradient was chosen depending on the polarity of fragments to be either one of the following:

HPLC-run normal (28 min): 0 min: 40% MeOH/60% phosphate buffer pH 2.3; 15 min: 85% MeOH/15% phosphate buffer pH 2.3; 20 min: 85% MeOH/15% phosphate buffer pH 2.3; 22 min: 40% MeOH/60% phosphate buffer pH 2.3; 28 min: 40% MeOH/60% phosphate buffer pH 2.3.

HPLC-run for polar fragments (28 min): 0 min: 10% MeOH/90% phosphate buffer pH 2.3; 15 min: 85% MeOH/15% phosphate buffer pH 2.3; 20 min: 85% MeOH/15% phosphate buffer pH 2.3; 22 min: 10% MeOH/90% phosphate buffer pH 2.3; 28 min: 10% MeOH/90% phosphate buffer pH 2.3.

### Protein Expression and Purification

DNA sequences of the AAK1 (121-471), CAMK1G (1-320), and DOT1L (1-416) were optimized for *E. coli* expression and synthesized by Thermo Fisher’s GeneArt service. Constructs were cloned into the pET24a_HLT vector (Boeckler et al., 2008). The pET15b_IDO1(1-435) was a generous gift from H. Sugimoto (Sugimoto et al., 2006). The thrombin cleavage site was changed to a TEV cleavage site by the Q5 Site directed Mutagenesis Kit from NEB.

#### AAK1 and CAMK1G

The pET24a_HLT_AAK1 or pET24a_HLT_CAMK1G construct was transformed into *E. coli* BL21 (DE3) pLysS cells with kanamycin (50 μg/ml) and chloramphenicol (34 μg/ml) as antibiotics. Cells were grown in 2xYT media to an OD_600_ = 0.4–0.6; at this time, 0.8 mM IPTG was added and the temperature was reduced to 18°C. Expression lasted overnight. Cells were separated by centrifugation for 30 min at 4,000 rpm in a J6-MI (Beckman-Coulter) centrifuge. The lysis buffer consisted of 50 mM HEPES, 500 mM NaCl, 10 mM imidazole, 5% (^V^/_V_) glycerol, and 0.5 mM TCEP with a pH of 7.5 (Sorrell et al., 2016). Cell lysis was done by ultrasound. The suspension was centrifuged in an Avanti J-30-I (Beckman-Coulter) at 18,500 rpm for 1 h. Two 5 ml HisTrap columns (Cytiva) were washed with 5 CVs of lysis buffer and eluted with 30% of the above-mentioned buffer in which the imidazole concentration was 300 mM. The eluted fractions were pooled, and TEV protease (1 mg/ml) was added for tag cleavage at an approximate 1:10 ratio. The reaction mixture was dialyzed overnight against the lysis buffer without imidazole and glycerol. The next day, a reverse nickel column run was performed to retain the cleaved HIS-tag and the HIS-tagged TEV protease. The flow through and washing steps were collected. This solution was concentrated and loaded onto a HiLoad 26/60 column filled with Superdex 75 pg (Cytiva) equilibrated with SEC buffer (50 mM HEPES, 300 mM NaCl, 5% (^V^/_V_) glycerol, 0.5 mM TCEP, pH = 7.5). The peak containing the protein of interest was collected, concentrated to 50 μM, flash frozen in liquid nitrogen, and stored at −80°C for further use. Proteins were identified by ESI-MS. The purity of the proteins was monitored by SDS-PAGE.

#### DOT1L

The pET24a_HLT_DOT1L construct was transformed in *E. coli* BL21 (DE3) pLysS cells. The expression and purification were performed as described for AAK1. The lysis buffer for DOT1L contained 20 mM TRIS, 500 mM NaCl, 10 mM imidazole, 5 mM β-ME, and 10% (^V^/_V_) glycerol with a pH of 7.8 (Min et al., 2003). The imidazole concentration in the elution buffer was 300 mM. The SEC buffer contained 20 mM HEPES, 200 mM NaCl, 1 mM EDTA, and 1 mM DTT with a pH of 7.8. The peak containing the DOT1L was collected, concentrated to 50 μM, flash frozen in liquid nitrogen, and stored at −80°C for further use. The protein was identified by ESI-MS. The purity of the protein was monitored by SDS-PAGE.

#### IDO1

The pET15bT_IDO1 construct was transformed into *E. coli* Rosetta 2 (DE3) pLysS cells (Novagen) with ampicillin (50 μg/ml) and chloramphenicol (34 μg/ml) as antibiotics. Cells were grown in TB medium to an OD_600_ = 0.5. The temperature was lowered to 18°C, and IPTG was added to final concentration of 0.5 mM. For heme incorporation 5-aminolevulinic acid was added to a final concentration of 0.5 mM. The expression lasted for 15 h. Cultures were centrifuged at 4,000 rpm for 30 min. The pellet was resuspended with lysis buffer (25 mM TRIS, 150 mM NaCl, 10 mM imidazole, 5 mM β-ME, pH of 7.4). After adding DNase and RNase, the suspension was sonified. The suspension was centrifuged at 18,500 rpm for 1 h. Afterwards, the supernatant was sterile filtered and loaded onto a Nickel-NTA Column (Cytiva) equilibrated in lysis buffer. The column was washed with 5 CV of the lysis buffer. Protein was eluted with 40% of elution buffer, which consisted of a 300 mM imidazole solution in lysis buffer. The fractions were pooled, TEV was added for cleavage, and the reaction mixture was left overnight. To subject the protein to a reverse nickel run, the protein was buffer exchanged into lysis buffer on a HiPrep Desalting 26/10 column (Cytiva). After the reverse nickel column, a 4 times excess of Hemin was added and left overnight. Before loading onto the ÄKTA system, the solution was sterile filtered. A SEC chromatography with 25 mM TRIS, 150 mM NaCl, and 5 mM β-ME was performed (Austin et al., 2004). The fractions containing IDO1 were pooled, concentrated to 50 μM, flash frozen in liquid nitrogen, and stored at −80°C. The protein was identified by ESI-MS. The purity of the protein was monitored by SDS-PAGE. To determine the heme incorporation, the absorption at 280 and 405 nm was measured and a quotient Q (Q = 280/405) was calculated with Q = 2.2 = 100% (Takikawa et al., 1988). A heme incorporation of >80% was obtained.

### Fragment NMR Screening

All fragments were stored at −20°C as solids in argon flushed glass bottles. For NMR screening, fragments were weighed and dissolved in DMSO-d_6_ to a stock concentration of 300 mM. Mother plates were prepared with a 100 mM concentration. From these plates, daughter plates were prepared with the fragments mixed in the plates to a concentration of 50 mM for each fragment. Daughter plates were opened a maximum of eight times. The plates were argon flushed and sealed with a HT121TS (HTA) and Foil Heat Seal (Biozym).

For NMR experiments, proteins were buffer exchanged to 100 mM sodium phosphate, 250 mM NaCl, and 2 mM MgCl_2_ with a pH of 7. For long time measurements, like the protein ^1^H NMR spectrum, the buffer exchange was performed twice to reduce residual buffer peaks. All experiments were performed on a Bruker Avance III HDX 700 instrument, equipped with a 5 mm Prodigy TCI cryo-probehead. For every protein, a standard ^1^H NMR experiment with 1 k scans and water suppression through presaturation was performed. The STD experiments adapted the pulse sequence published by Mayer and Meyer (1999), Mayer and Meyer (2001). The on-resonance frequency, which was determined from the ^1^H NMR spectra of the protein, was between 0.5 and 0.6 ppm; 40 ppm was used as the off-resonance frequency. For an interleaved acquisition of the on- and off-resonance, a pseudo-2D scheme was applied. The saturation was done by Gaussian pulses with a length of 50 ms and 60 dB of attenuation, with an interpulse delay of 1 ms leading to an excitation bandwidth of about 42 Hz. The screening was done with 16 scans of on- and off-resonance scans each, with a 3 s saturation time. The samples contained final concentrations of 20 µM protein, 10% (^V^/_V_) DMSO-d_6_, and 1 mM of each of the two fragments. A ^1^H-NMR experiment was performed for each compound to act as a reference spectrum in the STD experiments. All NMR experiments were carried out at 25°C.

### ITC

The protein was buffer exchanged into 50 mM HEPES, 100 mM NaCl, 2 mM MgCl_2_, and 1 mM TCEP at pH 7.4. The protein was concentrated to 50–100 µM, and DMSO was added to a final concentration of 5% (^V^/_V_). The fragments were dissolved in DMSO to yield a 100 mM stock solution, which was further diluted with buffer to yield a 5 mM solution also containing 5% (^V^/_V_) DMSO. A MicroCal iTC200 (Malvern) instrument was used with the measuring cell set to 25°C, while the cooling jacket was set to 15°C. After a 120 s initial delay following temperature equilibration, a first injection with 0.5 µl over 2 s was done. Nineteen injections with 2 µl over 4 s were performed every 180 s (Ruhmann et al., 2015). The measurement was conducted using a needle stirring speed of 1,000 rpm and a reference heat rate of 10 μcal/s. Experiments were aborted, when the measuring cell was unable to reach a heat rate greater than 9 μcal/s during equilibration. Affinities were averaged from duplicates.

### ESP-Plots

The calculations for the ESP plots and V_max_ assessment were performed using TURBOMOLE Version 7.4.1. A triple-ζ basis set (def2-TZVPP) was used throughout the study. MP2 calculations were done in combination with the resolution of identity (RI) technique and the frozen core approximation. The frozen core orbitals were defined using default settings by which all orbitals possessing energies below −3.0 au were considered core orbitals. The SCF convergence criterion was increased to 10^−8^ hartree for all calculations (Lange et al., 2019). Custom python scripts were used for plotting.

## Results

### STD-NMR Screening

**FIGURE 2:**
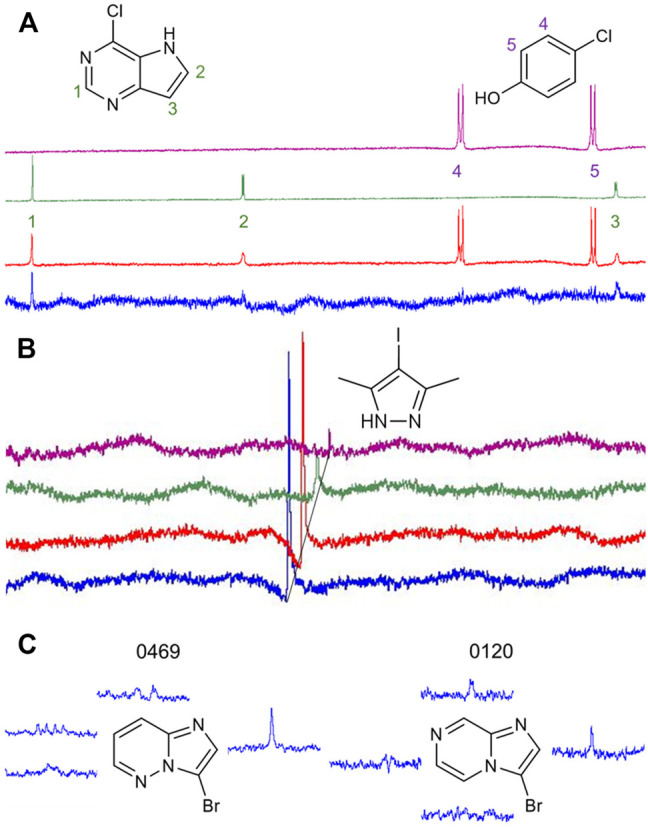
**(A)**: Comparison of the reference ^1^H NMR spectrum of the mixture of fragments 9595 and 4485 (red) with the spectrum of 9595 (green), 4485 (purple), and the difference spectrum with AAK1 (blue). While two of three protons of 9595 are visible in the difference spectrum, the spin system of 4485 (Signal 4 and 5) is obscured by the noise. **(B)**: Comparison of difference spectra of the same fragment mixture with AAK1 (blue), CAMK1G (red), IDO1 (green), and DOT1L (purple). All spectra show the same overlapping peak (CH_3_-signal of 0459), depicted with a 0.02 ppm offset between the spectra for improved visibility. **(C)**: Two examples of fragment hits binding to CAMK1G. NMR peaks were clipped and arranged in proximity to the corresponding protons. The fragments share similarity in structure, as well as in the spectrum. Only the singlet signal of the proton next to the halogen receives enough saturation to facilitate the classification of 0469 and 0120 as fragment hits. The vicinity of halogens to the strongest STD-signal was one criterion for fragment prioritization, because it indicates that the binding motif involving the halogen is more likely to engage in interactions with the protein binding site.

Our library comprises 191 fragments with at least one halogen present. Fifteen of these compounds contain the same halogen twice. No compound bears mixtures of different halogen atoms. Four proteins (AAK1, CAMK1G, IDO1, and DOT1L) were screened against this HEFLib using STD-NMR. A fragment was considered a hit if the peak intensity in the difference spectrum was three times higher than the local background noise. The factor of 3 sigma was chosen, by following the STD% concept (Begley et al., 2013). A reasonable balance between speed and clarity was achieved using mixtures of two fragments for NMR screening. Chemical compatibility and peak complementarity were assured for these mixtures. Samples containing more than two fragments were avoided to minimize the possible target competition and to decrease the risk of fragment interactions. Figure 2A depicts one of the hit spectra (blue) compared to the ^1^H reference NMR spectrum of fragment 9595 (green) and 4485 (purple). The ^1^H reference NMR spectrum of the mixture is shown in red for comparison. The singlet (Signal 1) belonging to fragment hit 9595 is clearly visible in the STD spectrum (blue), whereas the two doublets (Signal 2 and 3) are mostly hidden in the baseline noise. In contrast, none of the signals of fragment 4485 (Signal 4 and 5) are distinguishable from the baseline noise, although this spin system should be easier to detect based on the relative signal levels (red spectrum). This clearly indicates that only fragment 9595 binds to AAK1. As fragment mixtures can lead to competitive effects between their fragments, it is not advised to use the quantification of peak mixtures for hit ranking. In addition, the saturation transfer efficacy can be influenced by multiple factors, such as the relaxation time, the binding affinity and kinetics, the binding pose, and alternative conformations of the fragment (Aretz and Rademacher, 2019). Differences for the STD spectrum of the fragment hit 0459 with all four proteins are highlighted in Figure 2B.

Based on the previously discussed criteria for hit detection, the selection of a useful screening concentration is obviously a key factor for the resulting hit rate. We chose a medium screening concentration of 1 mM with a sensible balance between finding more weak affinity or less high affinity hits. For this fragment concentration, we consider a target hit rate of 10–20% as a reasonable goal. The hit rates in the IDO1 (16.2%) and CAMK1G (21.5%) screen comply well with this goal (Table 2). In retrospect, the concentration for the kinase screening (CAMK1G and AAK1) could have been lowered to reduce the number of weaker binders, as discussed before (Mashalidis et al., 2013). For DOT1L, a hit rate of only 4.7% was obtained. In this case, either the concentration or the number of scans could have been increased, to identify more, but weaker interactions. However, validation of hits in the millimolar affinity range is challenging. With our ITC approach and limited solubility of the fragments, we should not be able to detect such affinities with sufficient accuracy.

**TABLE 2:** Overall hits and hit rates for all targets. A matrix of unique and mutually binding fragment hits is appended. Percentages are provided with respect to the total number of hits for the target in the respective column.

	CAMK1G	AAK1	DOT1L	IDO1
Overall hits	41	57	9	31
Hit rate	21.5%	29.8%	4.7%	16.2%
CAMK1G	11	28 (49.1%)	4 (44.4%)	15 (48.4%)
AAK1	28 (68.3%)	20	8 (88.9%)	19 (61.3%)
DOT1L	4 (9.8%)	8 (14.0%)	0	4 (12.9%)
IDO1	15 (36.6%)	19 (33.3%)	4 (44.4%)	10

The overlap of fragment hits observed for both kinases (68.3 and 49.1%, respectively) might be attributed to the similar binding sites and overall architecture of the protein. However, we found a reasonable number of fragments binding to only one of the two kinases (11 fragments for CAMK1G and 20 fragments for AAK1). These fragments were especially interesting for the validation phase. The SAM-binding pocket of DOT1L and other allosteric binding pockets, which have been found (Mobitz et al., 2017), have distinct shape and binding features compared to the kinases. Hence, the low overlap between these targets. In contrast to the unselective fragment hits, the uniquely binding fragments may help to identify features that can be exploited for selectivity and, thus, contain more value for drug discovery. At least 10 fragments bind to CAMK1G, AAK1, and IDO1 in a unique fashion, whereas no unique fragment hits could be observed for DOT1L. This may be related to the overall low hit rate for DOT1L as well (Table 2).

**FIGURE 3:**
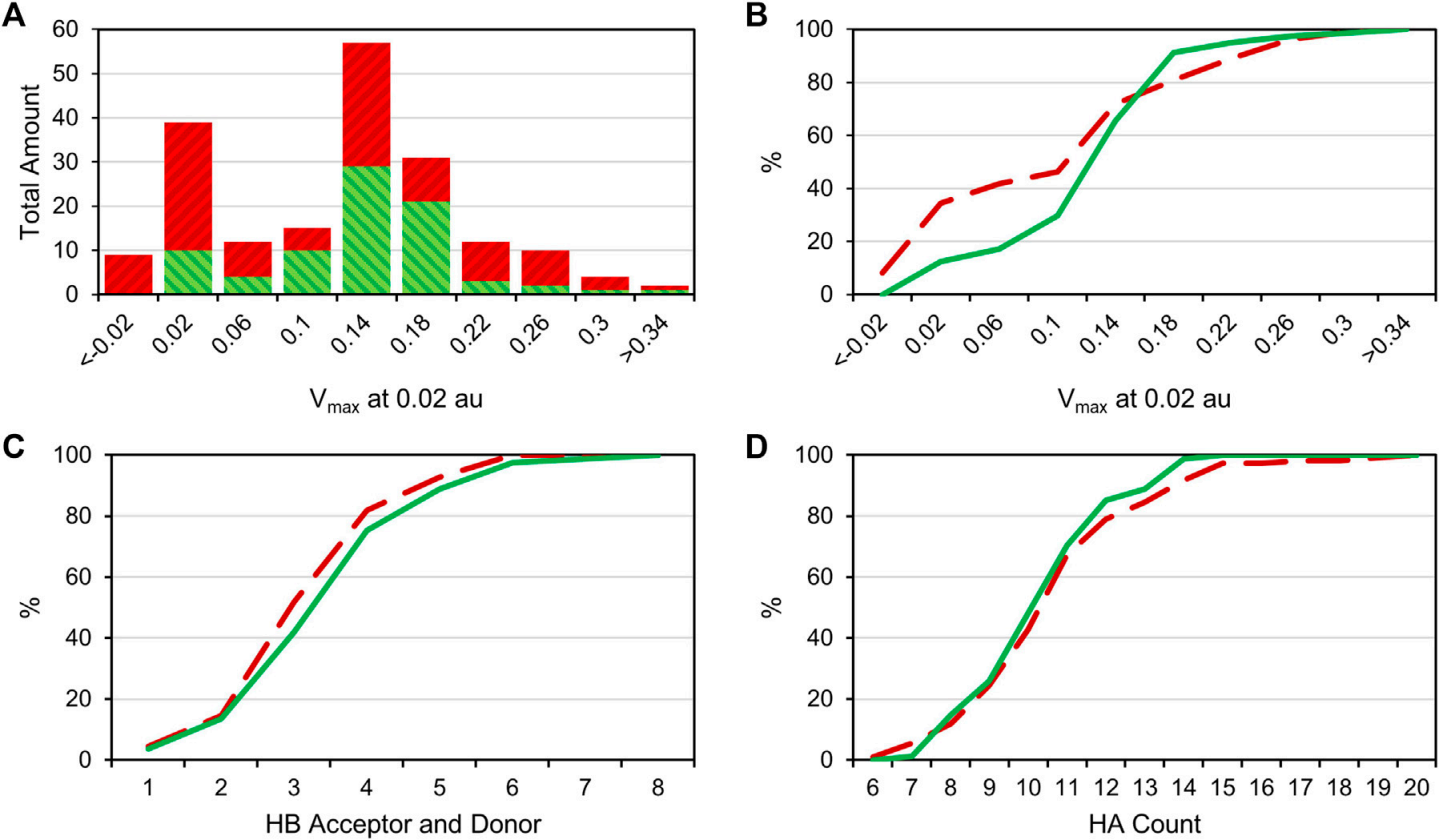
**(A)**: Bar plot comparing the V_max_-value at 0.02 au of all hit compounds (green) with the uneventful compounds (red). **(B)**: Cumulative distribution of the V_max_ data; green is the distribution of hit compounds, and red (dashed) is the distribution of uneventful compounds. A right shift of hits towards higher V_max_ values can be observed. **(C)**: Cumulative distribution of HB acceptors and donors. **(D)**: Cumulative distribution of (non-hydrogen) heavy atom (HA) number. No particular shift in the distribution is observable in panels C and D.

Comparing the different halogens in the library and the STD results, we see an extraordinary accumulation of hit events with iodine-containing fragments (Table 3). Although there are only 14 fragments bearing iodine, we found that nine of these fragments (64%) can interact with at least one of the target proteins. Forty different hits contain chlorine, which account for only 35% of all 114 chlorinated fragments. Fifty-one percent of all bromine fragments are found to be hits, placing the relative hit rate between chlorine and iodine. This is not unexpected, because the electron structure of the heavier halogens typically leads to the formation of increased σ-holes and, thus, stronger interactions. To investigate whether the ability to form stronger halogen bonds is related to the observed hit rates in our study, we have compared the maximum electrostatic potential (V_max_) on the isodensity surface of the halogen between fragment hits and non-binders. V_max_ can be used as a descriptor representing the size and accessibility of the σ-hole. It is known to indicate the strength of the resulting halogen bond (Heidrich et al., 2019; Lange et al., 2019). We find the average of V_max_ of all hit fragments compared to all fragments binding to none of the four targets (0.137 au vs. 0.113 au) to be significantly increased with *p* = 0.02 (Welch-t-test for unequal variances; see Supplementary Material S1). One obvious explanation for this observation is the higher representation of the heavier halides in the hit fragments and their naturally extended σ-hole (V_max_). On the other hand, this effect is diminished by the small number of iodine and bromine compounds compared to the chlorine fraction (114 chlorine, 63 bromine, 14 iodine). The distribution of V_max_-values compared between fragment hits and non-binders, as depicted in Figures 3A,B, illustrates and highlights the shift towards higher V_max_-values in the hit fraction. Neither number of hydrogen bond acceptors and donors (Figure 3C), nor heavy atom count (Figure 3D) shows a similar trend towards higher hit rates. This suggests that halogen bonding is a relevant factor in many binding events. In addition, HEFLibs focusing on V_max_-optimized fragments should provide higher hit rates in comparison to this diversity-optimized HEFLib.

**TABLE 3:** Comparison of relative hit rate by halogens type. Hit events summarize all four targets. Fragments with two halogens were counted only once. There are no fragments containing mixed types of halogens.

	Cl	Br	I
Fragments containing	114	63	14
V_max_ average (all fragments)	0.107	0.136	0.202
Hit events	72	48	18
of total compounds	63.2%	76.2%	128.6%
Hit compounds	40	32	9
of total compounds	35.1%	50.8%	64.3%
V_max_ average hits	0.117	0.144	0.209

**FIGURE 4:**
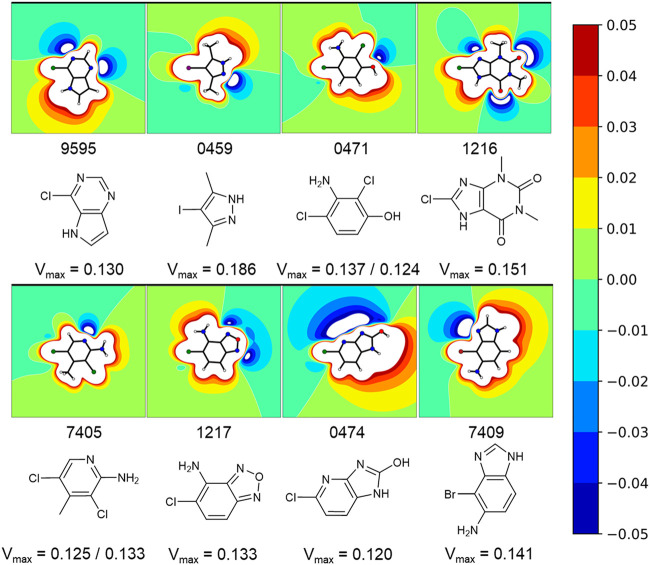
Exemplary fragments and their respective electrostatic potential (ESP) plots. The most common protomer at pH 7 was chosen for each fragment. The most likely protonation state at pH 7 was chosen using MOEs Protomer Generation (2019.0102). A reddish color marks electron deficient regions, whereas blues mark electron rich regions. The first of two values corresponds to the halogen atom oriented to the left. Depicted fragments vary within the magnitude of the σ-hole, as well as the halogen bonding motif. The ESP of fragment 0474 is split along the Cl-C-bond with a negative potential on the one side and a positive potential on the other side. A completely different halogen bonding motif can be seen for example in fragment 0459 or 1217.

A comparison of peak intensities or integrals of a STD spectrum is problematic. As mentioned before, fluctuations in a multitude of variables (e.g., relaxation time, varying binding sites, fragment binding thermodynamics, and kinetics) cause unpredictable shifts in the saturation transfer. To avoid misjudging the screening results, we did not rank STD hits by the amplification factor for example, as it was shown that the amplification factor and the binding affinity only correlate in very specific cases (Aretz and Rademacher, 2019). Hence, we prioritized fragments for validation on the basis of the diversity of the halogen bonding motif (Figure 4), which is influenced by the magnitude of the σ-hole and neighboring effects of positive and negative potentials, caused by heteroaromatic scaffolds and diverse substituents. The diverse electrostatic environments illustrate the plethora of possible interaction patterns. The examples also highlight that the σ-hole can be distorted in an asymmetric fashion (Figure 4, e.g., 9595, 0474) by heteroatoms or substituents in close proximity. The halogen bonding motif combined with a good STD signal of a hydrogen atom directly adjacent to the halogen was used as the primary criterion for hit prioritization (Figure 2C). In addition, we focused on fragments showing some target selectivity. Lastly, a few fragments were selected, because they bound to every screened protein. The last category was selected to test whether unspecific binding or other disruptions of the STD assay were present in the screening. These selection criteria were designed to improve the chances of identifying fragment binding modes that might rely on halogen bonding, while keeping the highly time and protein consuming ITC validation at a reasonable scale. Overall, 57 protein-fragment combinations were chosen for validation by ITC.

### Stability Assessment

In order to investigate whether chemical reactivity plays a role in the lack of selectivity of the observed pan-target hit 1234, we have used a GSH stability assay to assess its risk of covalent reactions with proteinogenic nucleophiles (mainly cysteines). Electron deficient, aromatic compounds are prone to nucleophilic attack in S_N_Ar-type reactions (Keeley et al., 2019). In our halogen-enriched fragments, the intrinsically present halogen atoms are modulated by electron withdrawing substituents to provide a variety of halogen bonding strengths, represented by different V_max_-values. However, halogens can be typical leaving groups and their reactivity in S_N_Ar type reactions is enhanced by electron withdrawing substituents. Thus, stronger halogen bond tuning could in principle lead to a decreased chemical stability.

**FIGURE 5:**
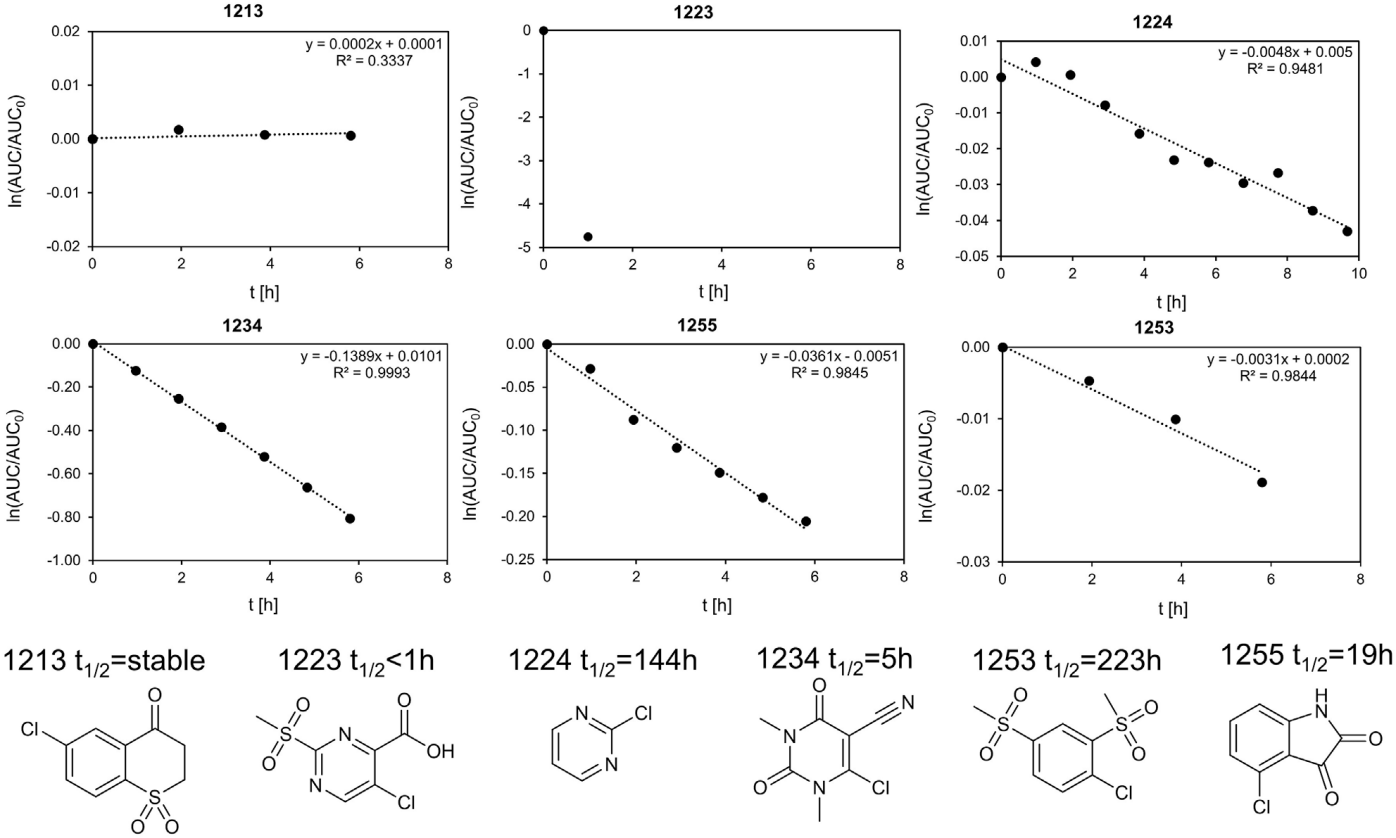
Stability assay for 1255 and 1234 with GSH. The linearized function of the AUC of the HPLC peak is plotted against the time. The extrapolated half-life is 19.2 and 5 h, respectively. We define half-lifes t_1/2_ > 20 h as sufficiently stable for fragment screening. As the half-life was measured at 40°C and the screening as 20°C, the stability of fragments with t_1/2_ > 20 h should be sufficient. Of the six fragments shown, the top row (fragments 1223, 1234, and 1255) is unstable, whereas the bottom row (fragments 1224, 1213, and 1253) was considered stable within our definition.

In total, six fragments were tested in a GSH stability assay as described previously (Keeley et al., 2019). Three fragments (1213, 1224, and 1253) showed half-lifes noticeably longer than 20 h, while three fragments (1223, 1234, and 1255) could not meet this stability criterion. With a half-life of 19.2 h, fragment 1255 was marginally outside our definition of chemical stability. As Isatins, like 1255, have been postulated as possible covalent inhibitors for carboxylesterases (Hyatt et al., 2007), the low reactivity with GSH indicates that it is unlikely to form unspecific covalent interactions in this study. Fragment 1234 is a potential PAIN compound with a half-life of 5 h (Figure 5) (Baell and Nissink, 2018). The third rather reactive fragment was 1223, which proved to be too reactive for the assay, so a half-life below 1 h could only be deduced. In addition, the literature describes fragment 1223 as a covalent fragment in a p53 FBDD study (Bauer et al., 2016). It should be noted that the observed reactivity of 1223 was not attributed to the halogen atom, but to the sulfone substructure. In our STD-based FBDD approach, it appears not unreasonable to also include potentially reactive fragments. Although this was not the design principle of the HEFLib, fragments unsuitable for halogen bonding could be included in a library focusing on covalent binding modes. Furthermore, the design of covalent fragment libraries has become a trend in FBDD (Keeley et al., 2019).

### ITC Validation

For the validation, ITC was chosen to incorporate a completely orthogonal principle of measurement of the binding event, compared to NMR. Millimolar affinities could not be resolved with a direct ITC approach, as the solubility of the fragments and to a lesser extent the protein consumption limited our study (Turnbull and Daranas, 2003). It bears some advantages over other techniques commonly utilized in fragment validation. The biggest advantage of ITC over, for example, SPR is the measurement in solution without any kind of labelling, avoiding potential interference of the binding event. Under the given experimental conditions, we experienced a limit of determination for accurate dissociation constants of approximately 800 µM. Thus, considering a mean heavy atom number of 11 for a typical fragment in our library, a ligand efficiency (LE) greater than 0.3 has to be observed in the validation (Table 4). Fragments with weaker affinity (i.e., lower LE) are usually considered disadvantageous for optimization. Thus, our validation strategy aimed for detecting affinities stronger than 800 µM and a LE > 0.3, generating hits that are deemed worthy for further investigation.

**TABLE 4:** Affinities and fragment metrics of all verified fragments with an accurate affinity estimation in the ITC. LE is defined as $\text{LE}=\frac{1}{N}\cdot 1.4\cdot -\log\left({\text{K}}_{\text{D}}\right)$ , LELP is defined as $\text{LELP}=\;\frac{\text{ logP}}{\text{LE}}$ , and K_D_-values were calculated from duplicates with a standard deviation < 15%.

Protein	Fragment	K_D_ [µM]	HA	LE	SlogP^a^	LELP
IDO1^b^	0459	>1,000	8		1.631	
IDO1	1216	390	14	0.34	−0.654	−1.92
CAMK1G	9595	200	10	0.52	1.611	3.11
CAMK1G	0459	190	8	0.65	1.631	2.51
CAMK1G^c^	7405	130	11	0.50	2.279	4.60
CAMK1G	7419	680	11	0.40	−0.608	−1.51
AAK1	9595	6	10	0.73	1.611	2.21
AAK1	0459	18	8	0.83	1.631	1.96
AAK1	0471	90	10	0.57	2.281	4.03
AAK1	0474	180	11	0.48	0.905	1.90
AAK1	7,409	6	11	0.67	1.908	2.86

a Calculated by *RDKit* (Wildman and Crippen, 1999)

b K_D_-value could not be determined reliable; data included for comparison.

c Fragment concentration reduced to 2 mM due to solubility.

The selected fragments from the STD screening had generally sufficient solubilities (>5 mM), and ITC proved to be a valuable tool for successful validation. Still, the unstable fragment 1234 (see *Stability Assessment*) became an insurmountable obstacle in an ITC assay, as the heat emission of side reactions exceeded the heat emissions typically associated with non-covalent binding by an order of magnitude.

Measurement with other proteins and fragments were inconspicuous. Fragments 0120, 0459, 1234, 1270, 7405, 7407, and 7420 were tested with the DOT1L. None of these could be validated with ITC. This may be attributed to affinities below detection level of the ITC (>1 mM). Fragment 0459 showcased less selectivity between the targets, but no unspecific binding, as we were able to determine affinities for the kinases CAMK1G and AAK1 of 190 and 18 µM (Table 4), respectively. Because STD-NMR is more sensitive by a factor of 10 compared to ITC, it was anticipated that not all STD-NMR hits could be successfully validated by ITC (Mashalidis et al., 2013).

**FIGURE 6:**
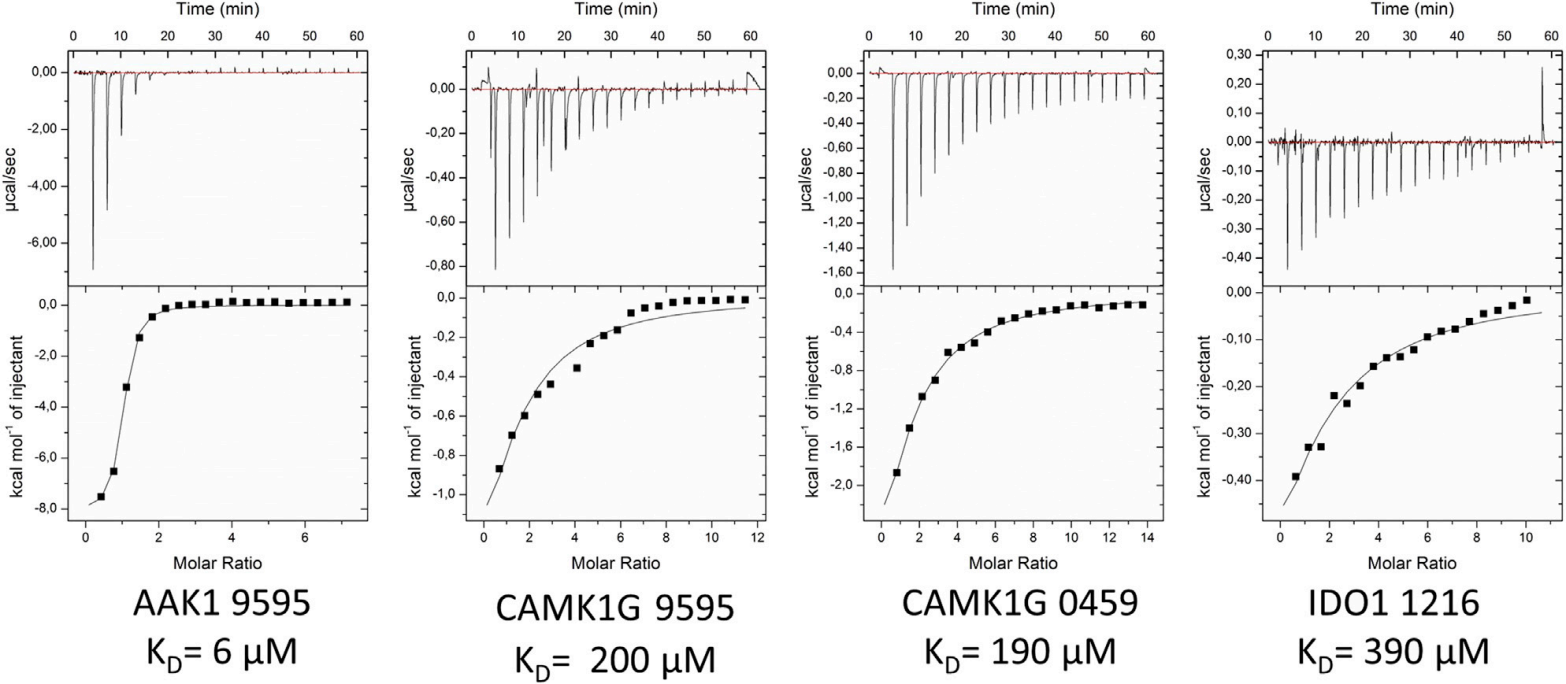
Exemplified ITC thermograms of four validated hits from the STD-NMR with at least one validated fragment per target. K_D_-values were estimated on duplicates. Standard deviation for measurements was below 15%.

The validation of IDO1 hits resulted in the identification of fragment 1216 as a micromolar binder. Figure 6 shows the thermograms of ITC measurements with a selection of verified fragments. With an affinity of 390 μM, the thermogram shows a clear trend in protein saturation. Due to the low protein saturation at these low affinities, the deviation in the curve fitting is of some concern. The overall standard deviation of both measurements remained within 15%. For fragment 0459, we were able to observe activity in the ITC data, which, however, was too low to derive reliable affinity determination. In particular, fragment 0459 was found to be a STD hit with every protein. We assume the dissociation constants of 0459 with DOT1L and IDO are beyond the limits of ITC. The STD screening of fragment 0459 with spin systems of three or more protons could be skewed as the detection of a large singlet is easier than a multiplet of a single proton. To test for a false positive based on aggregate formation or other disruption, we evaluated the affinity with all four proteins and could see different binding for every target (see Table 4). This indicates that unspecific binding or false positive STD spectra are highly unlikely.

For 12 fragments validated with CAMK1G, four K_D_-values could be determined (i.e., 0459, 7405, 7419, and 9595). One fragment (9605) showed peculiar heat emission, but the peaks were too small to fit a reasonable curve (Supplementary Figure S10). For fragment (0459), we were able to obtain a K_D_-value of 190 µM towards CAMK1G (Figure 6). Fragment 9595 binds with similar affinity and 7409 with an affinity of 130 µM. All three fragments show a ligand efficiency (LE) of over 0.4 (Table 4). In general, all verified fragments can be considered valuable starting points for optimization, as the lipophilicity corrected LE (LELP) lies within a favorable margin (Schultes et al., 2010; Tarcsay et al., 2012).

**FIGURE 7:**
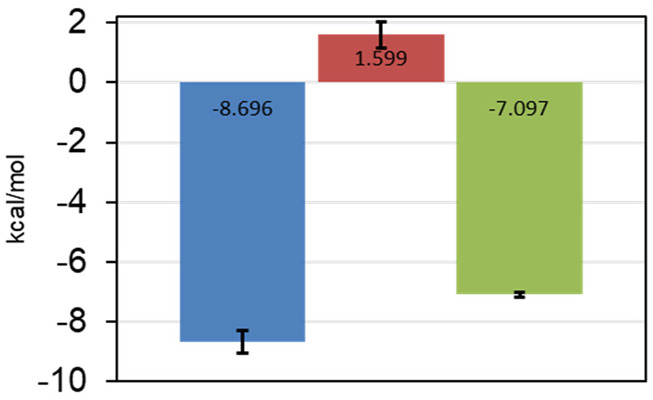
Thermodynamic parameters of 9595 determined from quadruplicate in the ITC. ΔH in blue, −TΔS in red, and ΔG in green. Standard deviations are shown as error bars. The fragment is mostly reliant on enthalpic effects for the binding mode.

The most promising results were obtained with AAK1, where we were able to verify five fragments. From these, one fragment (0474) was in the three-digit micromolar range, two fragments (0471 and 0459) showed a 10-fold better affinity, while the last two (9595 and 7409) seem to have low micromolar affinity (Table 4). For one of the two best fragments, a K_D_-value could be fitted, but compound 7409 shows a downward stepping baseline shift in the ITC, even if titrated into pure buffer, which we were unable to eliminate. This might be attributed to unspecific reactions with the buffer components or the cell wall, as no precipitation could be observed. Therefore, the affinity data of this fragment should be treated with caution. The best binding fragment was 9595 (Figure 6), with a K_D_ = 6 µM. In this affinity range, a higher C-value of 20–45 was obtainable (see Supplementary Material) and therefore, the thermodynamic parameters of the binding could be accurately determined (Figure 7). The enthalpic partition was calculated to −8.7 kcal/mol, and the entropic part was calculated as −1.6 kcal/mol from a quadruplicate. A high enthalpic partition combined with a low lipophilicity is considered as especially helpful in hit to lead optimization, as most lead molecules have a higher lipophilicity and optimization for entropic binding is considered faster (Tarcsay et al., 2012).

**FIGURE 8:**
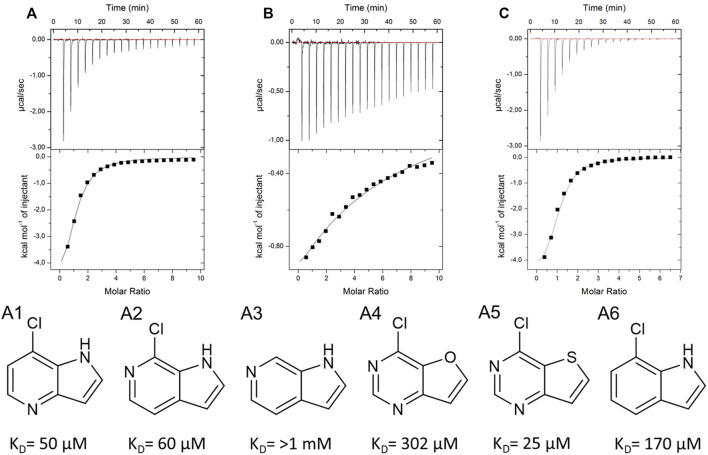
SAR compounds for the hit fragment 9595 with AAK. ITC plots show the raw data for the compounds A1 **(A)**, A3 **(B)**, and A5 **(C)**.

As a crystal structure of the AAK1 could not be obtained, we measured a small set of analogs to characterize structure activity relationships (Figures 8A–C). We could show that the pyrrole nitrogen was essential for the binding (Figure 8C A5), which, like removing the halogen, completely abolished the affinity (Figure 8B A3). Removal of either pyrimidine nitrogen atom resulted in a seven-fold decrease in affinity (compare A1 and A3). 7*H*-pyrrolo[2,3-*d*]pyrimidines are known as kinase inhibitors; therefore, we tested this fragment with three other kinases (DYRK1a, JNK2, and JNK3). A standard hinge binding mode should result in binding affinity of similar magnitude with most kinases. As it turns out, fragment 9595 shows a low micromolar affinity only with the AAK1; the other four kinases were bound with at least a 10-fold decreased affinity (CAMK1G: K_D_ = 210 μM, DYRK1a: K_D_ = 210 μM, JNK2: K_D_ = 67 μM, JNK3: K_D_ = 130 µM). The literature of kinase inhibitors uses 4-chloro-5*H*-pyrrolo[3,2-*d*]pyrimidine extensively as a synthetic building block (Oguro et al., 2010; Ishikawa et al., 2011; Gangjee et al., 2012), but it has not been described as a binder by itself. Consequently, we hypothesize a different binding mode than the standard hinge motif, with the chlorine as a major contributor to affinity.

## Conclusion

With our now established screening protocol, we were able to identify and validate a divers set of fragments binding to various classes of proteins. Within the screening protocol, we have utilized electrostatic potential calculations to prioritize initial hit fragments with higher halogen bonding capabilities. It should be noted that even in the primary hit results, an accumulation of fragments with higher V_max_-values was observed. Other factors, such as the number of hydrogen bonds or the number of heavy atoms, were not found to be enriched in the primary hits. The enrichment of high V_max_-values could lead to potentially reactive fragments. However, the validation by ITC should be able to distinguish between covalent or noncovalent binding modes.

Based on this library characterization, we will continue to develop the library in a number of ways. Firstly, we will aim to extend our library with additional fragments featuring a strongly tuned σ-hole of the halogen. The correlation between V_max_ and the number of hit fragments, combined with the overabundance of iodine within these hits, leads us to the conclusion that an increase in iodine-containing fragments will lead to improved HEFLib characteristics. Overall content of iodine in the HEFLib is so far limited, because of reduced commercial availability and higher costs of suitable iodinated fragments.

The overabundance of hits was further prioritized and subjected to hit validation by ITC. Due to the reduced sensitivity of ITC compared to STD-NMR, only fragments with desirable LE were validated. The validation of kinase hits was remarkably more successful than IDO1 and DOT1L. Ten fragments with good ligand optimization parameters could be validated, as their lipophilicity was rather favorable, leading to a remarkably good LE of up to 0.83 and low or even negative LELP-values. In part, the high solubility and low HA count is responsible for these good metrics. The best binding fragment 9595 with the AAK1 was further characterized. The SAR obtained by several close analogs highlighted the importance of chlorine in the binding mode. Although 4-chloro-5*H*-pyrrolo[3,2-*d*]pyrimidine has been described as a synthetic building block for various kinase inhibitors, the binding capacities of the fragment has not been evaluated before. Due to the importance of the chlorine atom for maintaining the affinity, we assume that it shows a novel binding motif, which has not been described before, featuring either a halogen bond or multiple polar interactions. This is supported by the preference of the fragment towards AAK1 with a factor of 10–30 compared to the other five kinases we have tested. The hits validated by ITC will be further explored either in an SAR campaign by in house generated compounds or by X ray crystallography. Especially, the CAMK1G and IDO1 fragments could not be addressed adequately so far. In addition, we will focus our attention to the crystallographic aspect of FBDD to elucidate the binding modes of 9595 and other fragments.

## Data Availability

The original contributions presented in the study are included in the article/Supplementary Material. Further inquiries can be directed to the corresponding author.
