# Supplementary material for: Quadruple Target Evaluation of Diversity-Optimized Halogen-Enriched Fragments (HEFLibs) Reveals Substantial Ligand Efficiency for AP2-Associated Protein Kinase 1 (AAK1)
Source: Front Chem. 2022 Feb 2;9:815567. doi: 10.3389/fchem.2021.815567 (PMC8847695; doi:10.3389/fchem.2021.815567)
Supplement: Supplementary file 2 [file DataSheet1.DOCX]

Supplementary Material

Diversity driven Characterization of HEFLibs reveals substantial ligand efficiency with AAK1 hits

Marcel Dammann^1^, Markus Kramer^2^, Markus O. Zimmermann^1^, Frank M. Boeckler^1, 3, *^

^1^Lab for Molecular Design & Pharm. Biophysics, Institute of Pharmaceutical Sciences, Department of Pharmacy and Biochemistry, Eberhard Karls Universität Tübingen, Tübingen, Germany

^2^Institute of Organic Chemistry, Eberhard Karls Universität Tübingen, Tübingen, Germany

^3^ Center for Bioinformatics Tübingen (ZBIT), Eberhard Karls Universität Tübingen, Tübingen, Germany.

*** Correspondence:**Frank M. Boeckler
frank.boeckler@uni-tuebingen.de

# Supplementary Figures


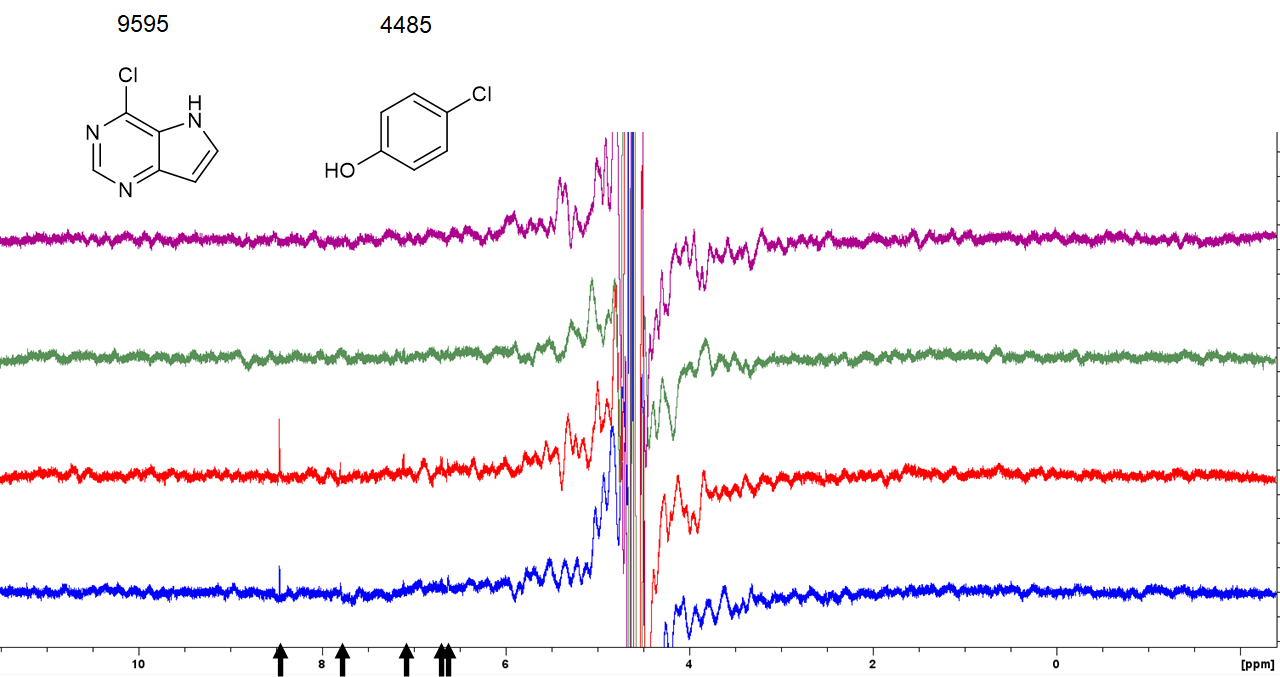


**Figure S1.** STD-NMR spectra of fragment 9595 and 4485. The spectra are superposed with AAK1 (blue) at the bottom, followed by CAMK1G (red), IDO1 (green), and DOT1L (purple) at the top. Arrows indicate hit signals.


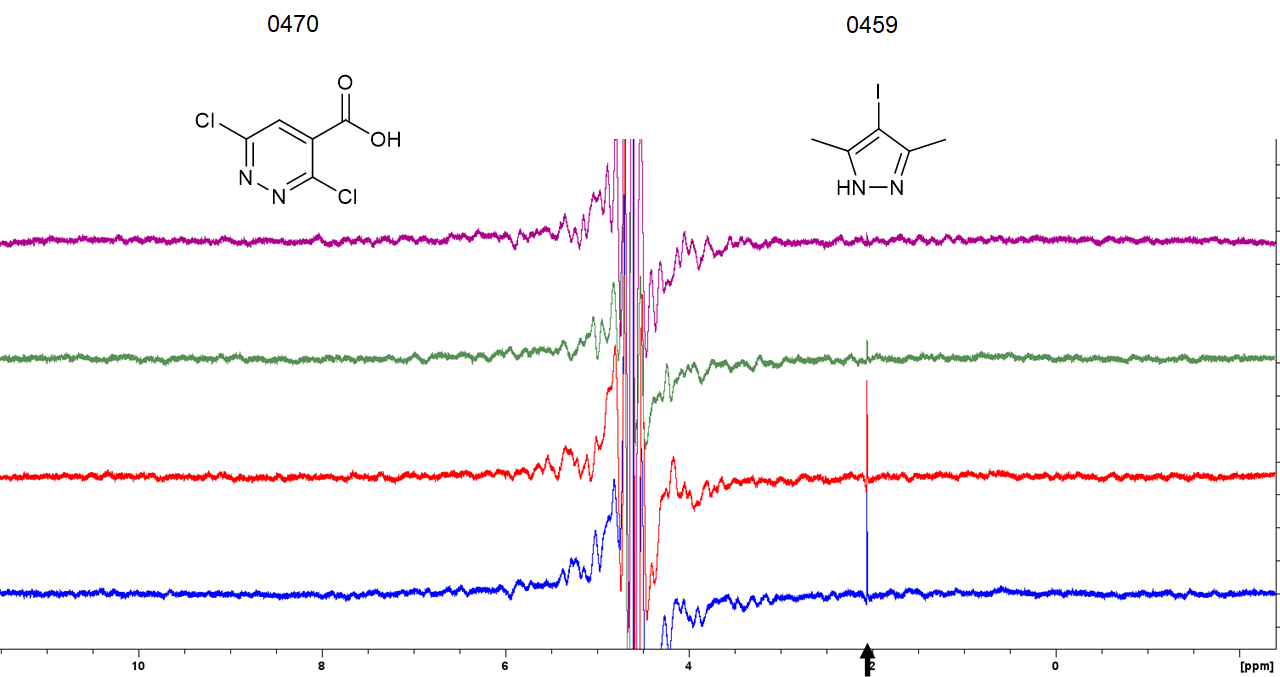


**Figure S2.** STD-NMR spectra of fragment 0459 and 0470. The spectra are superposed with AAK1 (blue) at the bottom, followed by CAMK1G (red), IDO1 (green), and DOT1L (purple) at the top. Arrows indicate the hit signals.


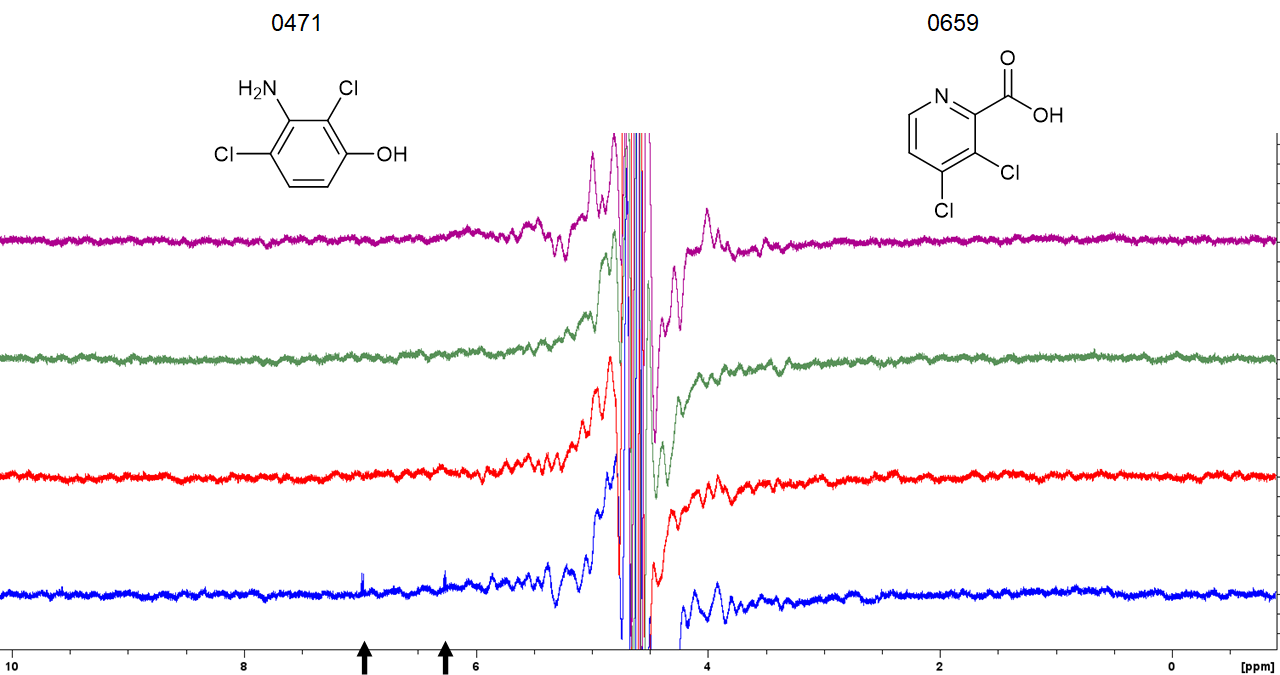


**Figure S3.** STD-NMR spectra of fragment 0471 and 0659. The spectra are superposed with AAK1 (blue) at the bottom, followed by CAMK1G (red), IDO1 (green), and DOT1L (purple) at the top. Arrows indicate the hit signals.


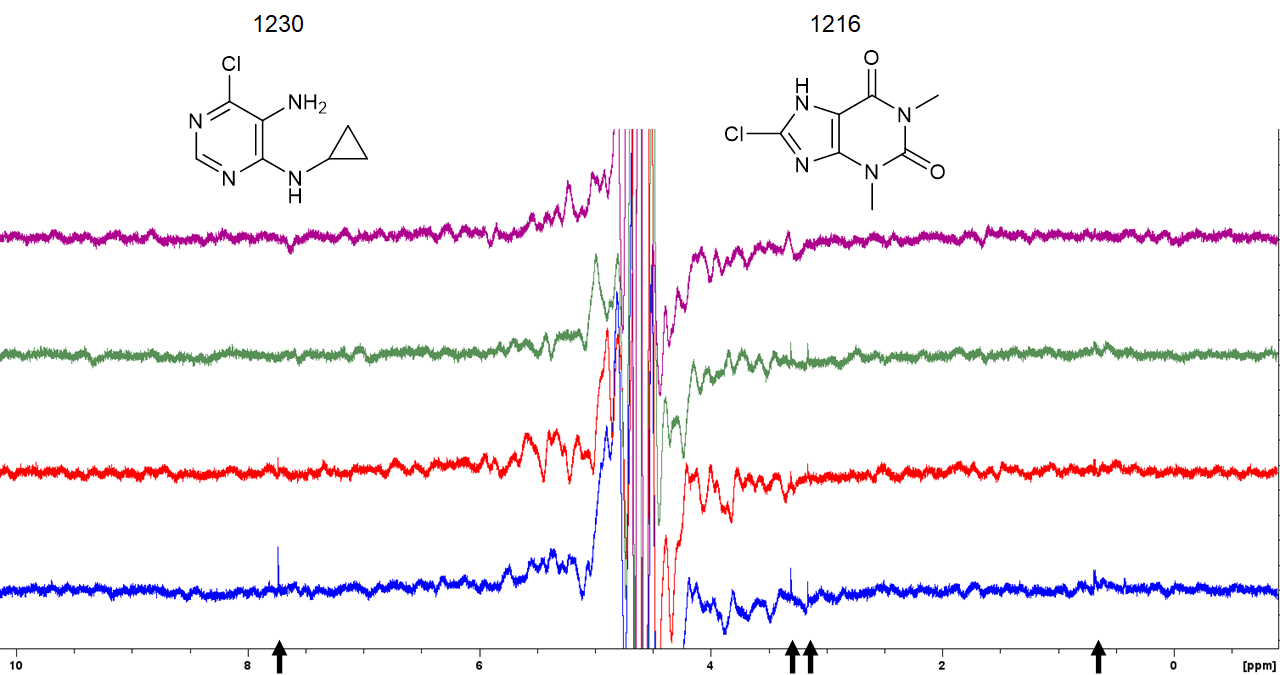


**Figure S4.** STD-NMR spectra of fragment 1216 and 1230. The spectra are superposed with AAK1 (blue) at the bottom, followed by CAMK1G (red), IDO1 (green), and DOT1L (purple) at the top. Arrows indicate the hit signals.


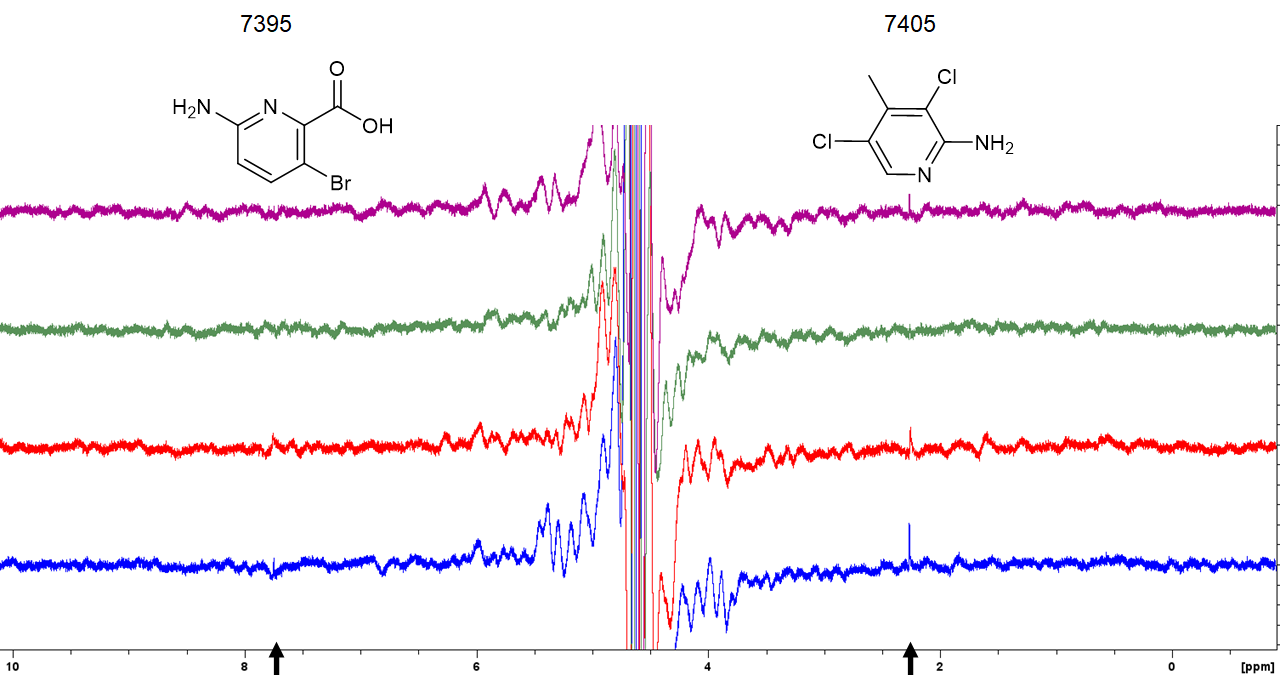


**Figure S5.** STD-NMR spectra of fragment 7395 and 7405. The spectra are superposed with AAK1 (blue) at the bottom, followed by CAMK1G (red), IDO1 (green), and DOT1L (purple) at the top. Arrows indicate the hit signals.


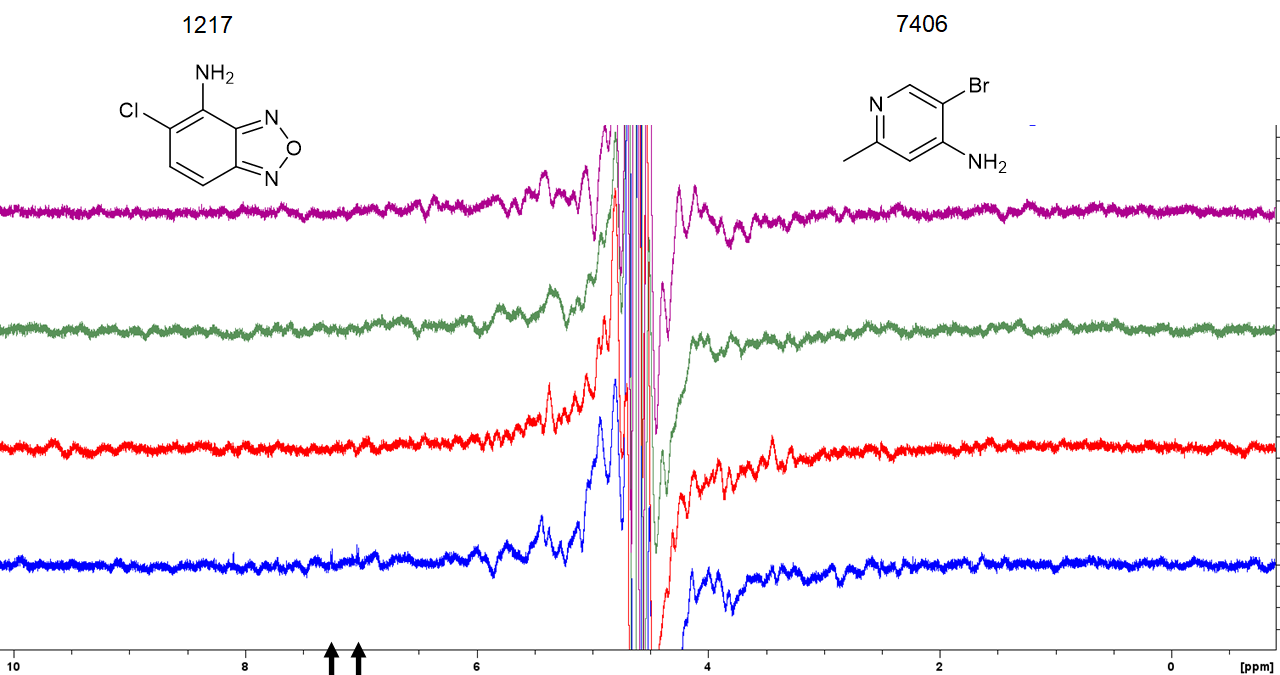


**Figure S6.** STD-NMR spectra of fragment 1217 and 7406. The spectra are superposed with AAK1 (blue) at the bottom, followed by CAMK1G (red), IDO1 (green), and DOT1L (purple) at the top. Arrows indicate the hit signals.


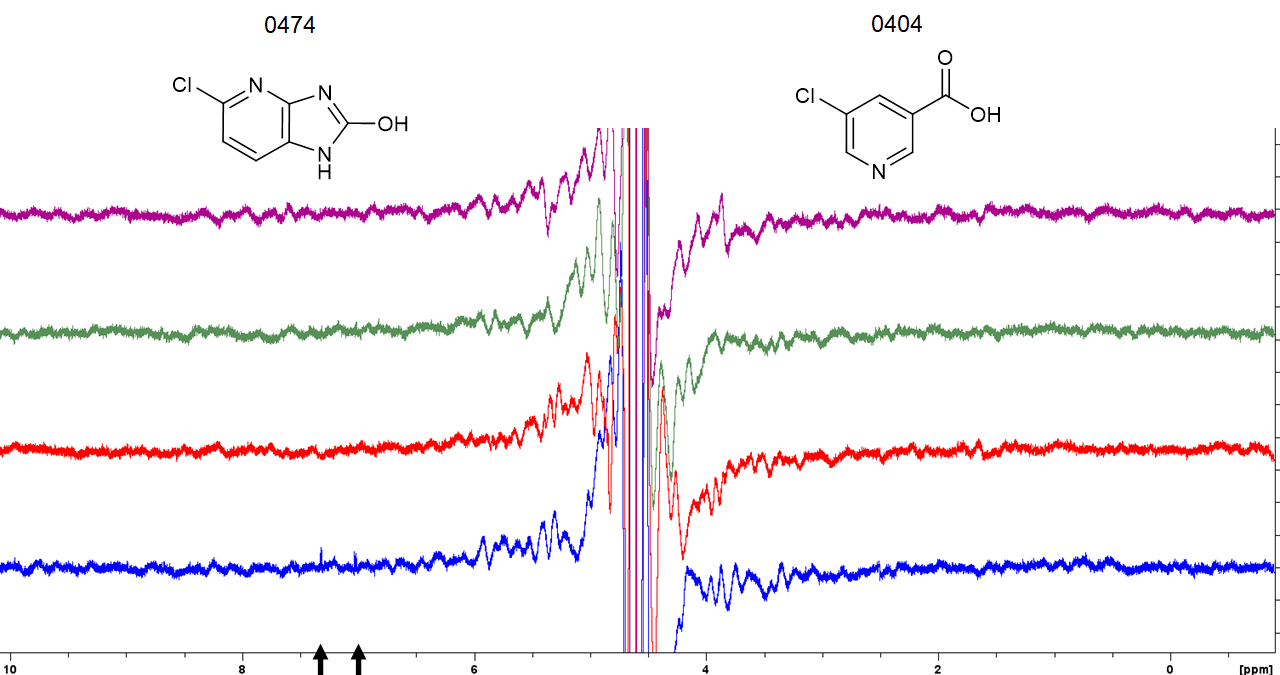


**Figure S7.** STD-NMR spectra of fragment 0404 and 0474. The spectra are superposed with AAK1 (blue) at the bottom, followed by CAMK1G (red), IDO1 (green), and DOT1L (purple) at the top. Arrows indicate the hit signals.


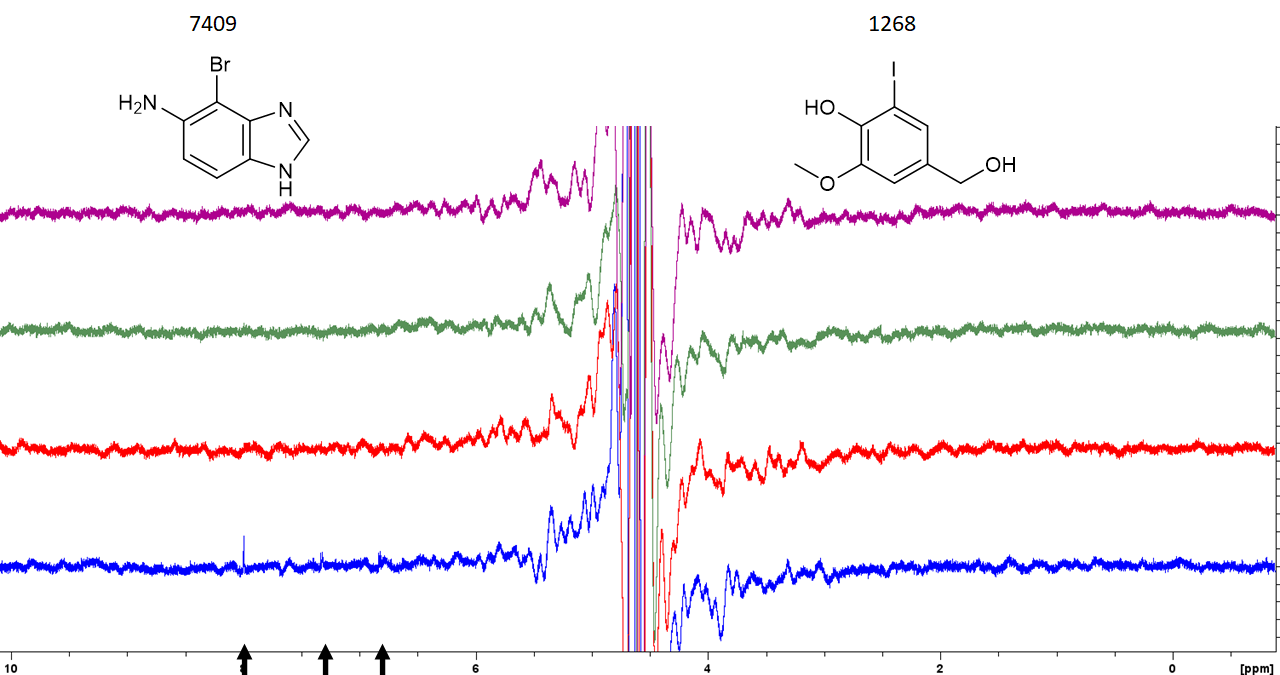


**Figure S8.** STD-NMR spectra of fragment 1268 and 7409. The spectra are superposed with AAK1 (blue) at the bottom, followed by CAMK1G (red), IDO1 (green), and DOT1L (purple) at the top. Arrows indicate the hit signals.


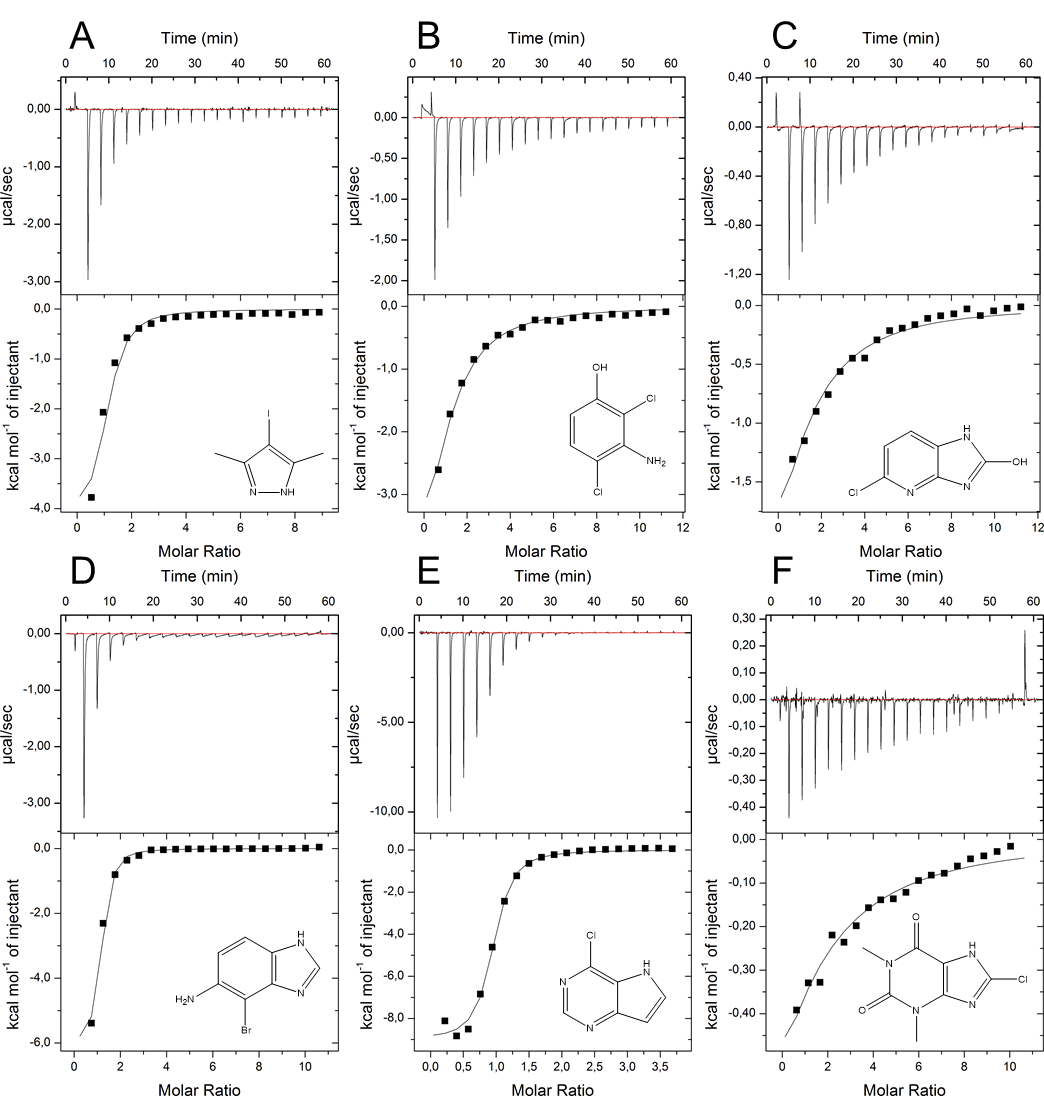


**Figure S9.** Thermograms of hit fragments 0459, 0471, 0474, 7409, 9595 with AAK1 (A-E) and F: IDO1 with 1216.


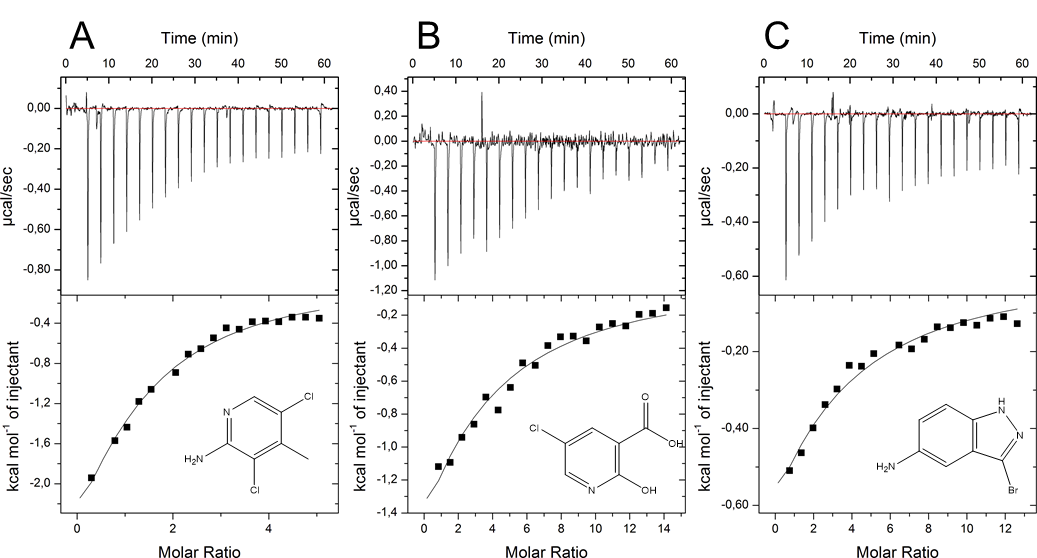


**Figure S10.** Thermograms of hit fragments 9605, 7419, and 7405 with CAMK1G (A-C).


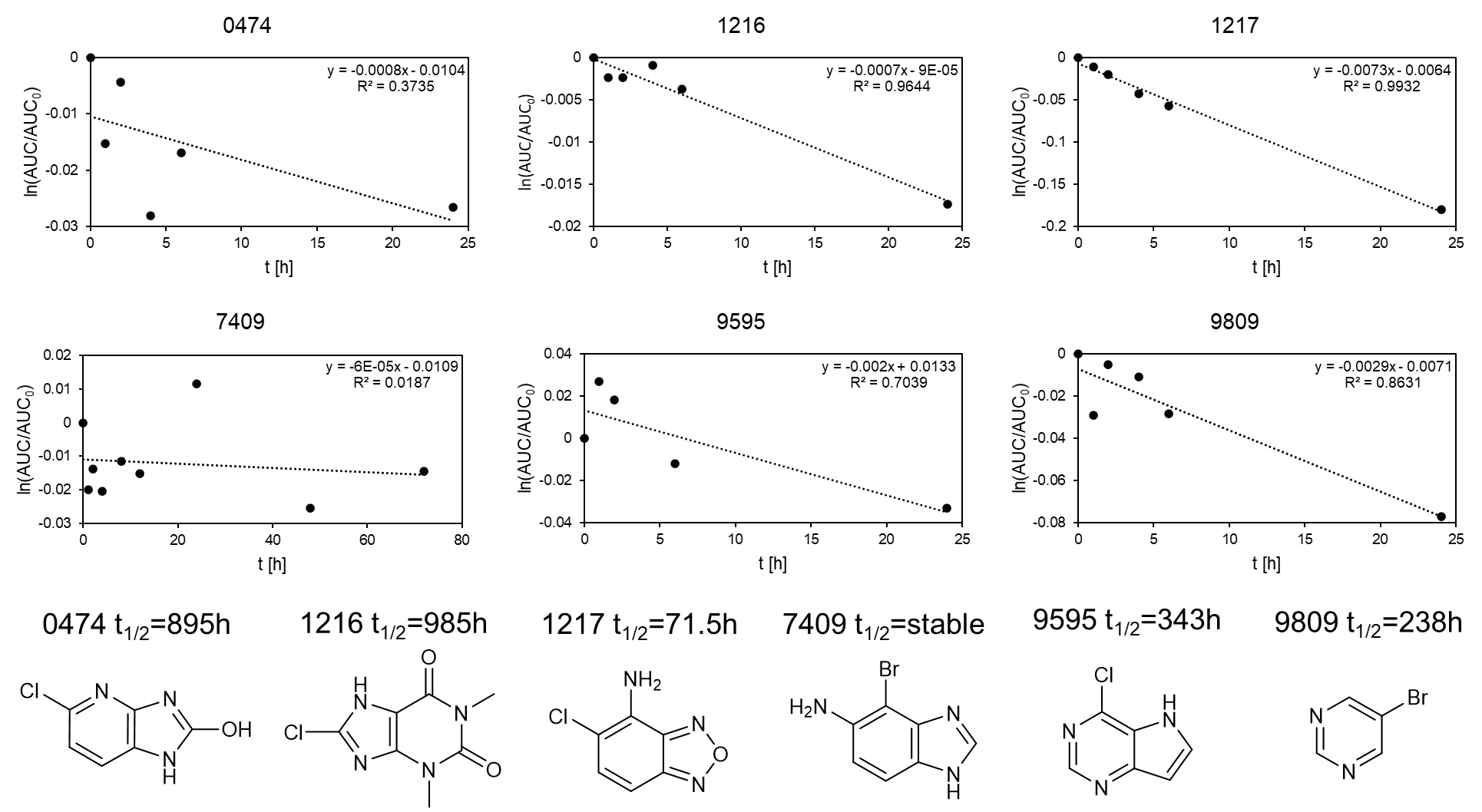


**Figure S11.** Stability assay for 0474, 1216, 1217, 7409, 9595, and 9809 with GSH. The linearized function of the AUC of the HPLC peak is plotted against the time. All measured compounds are regarded as sufficiently stable.
